# The Effect of Immunosuppressive Treatment on Torque Teno Virus Load in Lung Transplant Recipients: A Preliminary Study

**DOI:** 10.3390/v17030438

**Published:** 2025-03-19

**Authors:** Marek Ochman, Dagmara Galle, Anna Kowal, Magdalena Królikowska, Fryderyk Zawadzki, Anita Stanjek-Cichoracka, Anna Łaszewska, Elżbieta Chełmecka, Tomasz Hrapkowicz

**Affiliations:** 1 Department of Cardiac, Vascular and Endovascular Surgery and Transplantology, Medical University of Silesia in Katowice, 41-800 Zabrze, Poland; ochmann@wp.pl (M.O.); kowala124@gmail.com (A.K.); madziakrolikowska@gmail.com (M.K.); thrapkowicz@sum.edu.pl (T.H.); 2 Department of Pulmonology and Internal Medicine, Wielkopolska Centre for Pulmonology and Thoracic Surgery, 60-569 Poznań, Poland; zawadzkifryderyk@gmail.com; 3 Department of Biophysics, Faculty of Pharmaceutical Sciences, Medical University of Silesia in Katowice, 41-200 Sosnowiec, Poland; anita.stanjek@sum.edu.pl; 4 Laboratory of Transplant Immunology, Silesian Centre for Heart Diseases, 41-800 Zabrze, Poland; alaszewska@sccs.pl; 5 Department of Medical Statistics, Faculty of Pharmaceutical Sciences, Medical University of Silesia in Katowice, 41-200 Sosnowiec, Poland; echelmecka@sum.edu.pl

**Keywords:** TTV, lung transplantation, molecular biomarker, immunotherapy biomarker

## Abstract

After transplantation, systematically monitoring and assessing the risk of transplanted organ rejection is crucial. Current methods involving immunosuppressant monitoring, the assessment of organ function, and biopsies are insufficient for predicting rejection. However, regular determination of torque teno virus (TTV) load after transplantation may prove to be a useful parameter for monitoring immunosuppression efficacy. Therefore, we aimed to evaluate TTV load in patients before and after lung transplantation and the kinetics of TTV growth in relation to immunosuppression strength. We included 14 patients (mean age: 49.4 ± 14.0 years) undergoing lung transplantation and determined TTV copy numbers using the commercial ARGENE TTV-R-GENE kit from BioMerieux from the day of transplantation to 180 days post-transplantation. We also developed an empirical immunosuppression unit scale to calculate immunosuppression strength. We observed an average positive correlation between log_10_ TTV and immunosuppression strength, with significant increases in log_10_ TTV depending on the duration of immunosuppression. These results indicate the potential of TTV as a new parameter to assess the possibility of transplanted organ rejection.

## 1. Introduction

In patients undergoing solid organ transplantation, including lung transplantation (LuTx), maintaining adequate levels of immunosuppressive drugs poses a significant challenge. Both excessive and insufficient immunosuppression can lead to numerous complications for organ recipients. Patients who are over immunosuppressed have an increased risk of adverse immunosuppressive drug reactions, infections, metabolic, hematologic, and gastrointestinal complications, etc. Conversely, insufficient immunosuppression may result in acute and chronic organ rejection, which is a leading cause of death after LuTx.

The standard post-transplant immunosuppressive treatment involves a multidrug regimen, including calcineurin inhibitors (CNIs), antiproliferative drugs, mTOR inhibitors, and costimulation inhibitors, e.g., belatacept or abatacept (a CTLA-4 Ig fusion protein that blocks the CD28-CD80/CD86 pathway) and emerging agents like iscalimab (anti-CD40 monoclonal antibody), with the addition of glucocorticosteroids. In lung transplantation, maintenance therapy typically includes a CNI (either cyclosporine or tacrolimus), mycophenolic acid, and glucocorticosteroids. The prevailing method to monitor immunosuppression involves measuring drug concentrations in the patient’s blood and adjusting dosages to maintain therapeutic levels. However, the impact of dose fluctuations on individual patients cannot be predicted using this method. Gradual deterioration of graft function may not be detected in time to prevent rejection [1].

A reliable method to accurately determine the strength of immunosuppression remains elusive, suggesting that even therapeutic doses may not effectively prevent organ rejection [2]. Consequently, research is increasingly focused on identifying molecular biomarkers to predict lung transplant rejection. One promising avenue is linking torque teno virus (TTV) load to immune system activity, which could serve as a valuable tool for evaluating immunosuppressive treatment efficacy, potentially preventing rejection while minimizing drug-related side effects.

## 2. Materials and Methods

### 2.1. Statistical Analysis

The results for quantitative variables are presented as mean and standard deviation (for data with normal distribution) or as median and quartiles (for data with non-normal or skewed distribution): Me (Q1–Q3)—median (lower–upper quartile). A review of data distribution normality was performed with the Shapiro–Wilk test and quantile plot. Whenever necessary, the normality of variables was improved using a logarithmic transformation. According to the data distribution, Student’s *t*-test for independent samples was used. To determine the relationship between quantitative variables, ordinary least squares (OLS) regression was performed. Statistical significance was set at *p* < 0.05, and all tests were two-tailed. Statistical analysis was performed using Statistica v. 13.3.0 (TIBCO Software Inc., Tulsa, OK, USA).

### 2.2. Immunosuppression Regimen and Sampling Methods

Of the patients, 93% (13 patients) received an immunosuppressive treatment regimen consisting of tacrolimus, mycophenolate mofetil, and encortolone; 1 patient received dual-drug maintenance immunosuppression—tacrolimus and encortolone—due to the myelosuppression adverse effect of mycophenolic acid and CMV infection. In the study group, the amount of the TTV load was determined from the day of transplantation and 180 days post-transplantation, and the strength of immunosuppression was determined. Plasma was collected using EDTA as an anticoagulant via an intravenous catheter before transplantation and subsequently every 30 days for a period of 6 months. The collected blood sample was centrifuged, and the supernatant in the form of plasma was drained and frozen at −20 °C until the tests were performed.

### 2.3. TTV Measurement and Immunosuppression Assessment

DNA was isolated semi-automatically using the EasyMag system, and TTV determinations were performed using the ARGENE TTV-R-GENE kit from BioMerieux (Marcy-l’Étoile, France). As TTV viremia is associated with the intensity of immunosuppression, an empirical unit scale was developed to calculate immunosuppression strength. Each administered drug at a specific dose was assigned 1 unit of immunosuppression: 500 mg mycophenolate mofetil, 5 mg prednisone, and 2 mg sirolimus. For tacrolimus assessment, blood concentrations were used, as they are likely a more reliable parameter for evaluating the individual effect of the drug. One unit of immunosuppression corresponds to the normal blood concentration of tacrolimus; decreased values were assigned a value of 0, whereas increased values were assigned 2 units of immunosuppression. Linearity of the standard curve was observed between log1.8 and log9.0.

## 3. Results

### 3.1. Study Participants and Clinical Profiles

This study involved 14 patients undergoing lung transplantation at the Silesian Center for Heart Diseases in Zabrze in 2022. The group comprised eight women and six men with a mean age of 49.9 ± 14.0 years. Statistically, the age did not significantly differ between the men and women (*p* = 0.650, women: 47.9 ± 5.2 years, men: 51.5 ± 13.3 years). Diagnoses within the group included interstitial lung disease (ILD) in half of the patients, COPD in four (29%), primary pulmonary hypertension (IPAH) in two (14%), and CF in one patient (7%). None of the recipients (n = 14) experienced rejection during follow-up.

### 3.2. Strong Positive Correlation Between Time Since Transplantation and log_10_ TTV Levels

In the study group, we observed a significant strong positive correlation between time since transplantation and log_10_ TTV (r = 0.828, *p* < 0.001). Significant changes in log_10_ TTV were observed depending on the time elapsed post-transplantation (*p* < 0.05).

### 3.3. Systematic Increase in log_10_ TTV Without Corresponding Changes in Immunosuppression Strength

**Figure 1:**
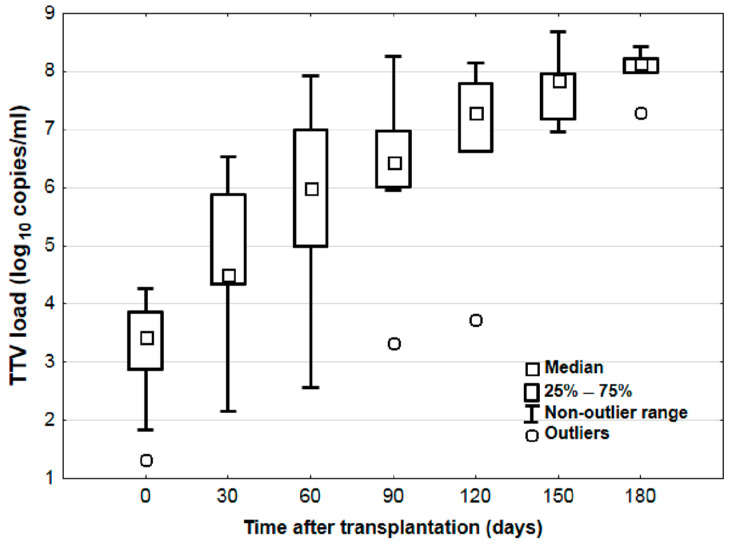
Changes in torque teno virus (TTV) as log_10_ viral copies/mL at different time points after lung transplantation.

**Figure 2:**
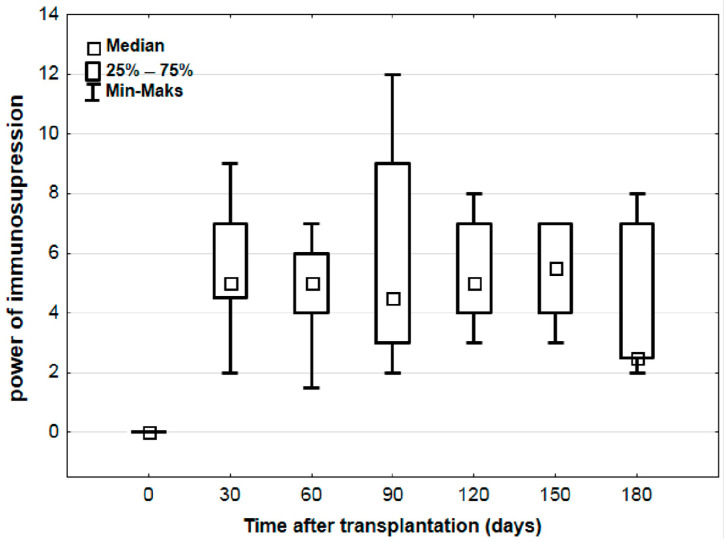
Changes in immunosuppression intensity over time after lung transplantation.

The results indicated a systematic increase in the log_10_ TTV parameter until approximately 150 days post-transplantation, reaching log8.3 ± 0.3 (Figure 1). The calculated strength of immunosuppression for patients ranged from 2 to 12 units. No statistically significant differences in the strength of immunosuppression were observed depending on the time post-transplantation (*p* = 0.533) (Figure 2).

### 3.4. Positive Correlation Between log_10_ TTV Levels and Immunosuppression Strength

**Figure 3:**
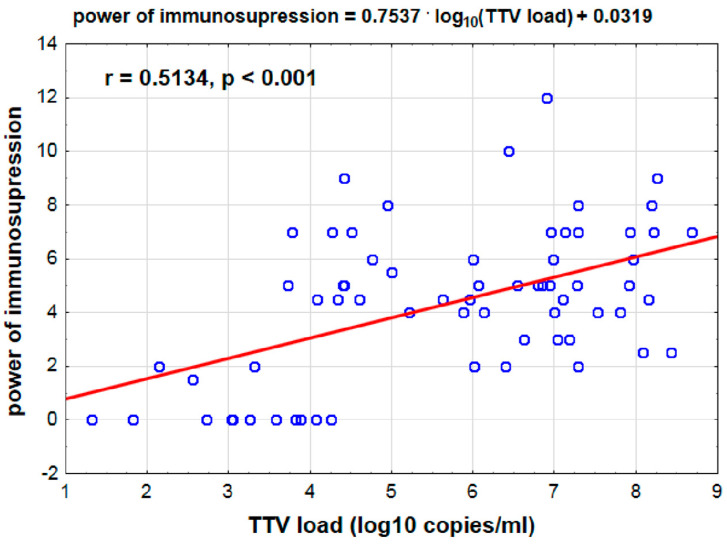
Relationship between immunosuppression and TTV viremia. Red line indicates OLS linear fit.

An average positive correlation was found between the time since transplantation and the strength of immunosuppression (r = 0.406, *p* < 0.001), similar to the log_10_ TTV parameter. Additionally, an average positive correlation was observed between log_10_ TTV and the strength of immunosuppression (r = 0.513, *p* < 0.001). Higher log_10_ TTV values corresponded to greater immunosuppression strength (Figure 3).

### 3.5. No Correlation Between TTV Levels and Donor-Specific Antibodies or CMV Infection

In eight patients, donor-specific antibodies were detected approximately one month after transplantation. In three patients, cytomegalovirus (CMV) infection was confirmed within 1 to 3 months after transplantation. No correlation was found between changes in TTV levels and the presence of donor-specific antibodies or CMV infection.

## 4. Discussion

A considerable challenge in transplantation is maintaining a balance between immunosuppressive drug therapy and the complications of administered treatment. There are few tools in modern diagnostics to accurately determine optimal and personalized immunosuppressive therapy [3]. Currently, predictions of immunosuppressive drug concentrations rely solely on pharmacokinetics and do not predict clinical outcomes [4]. The most common method to assess immunosuppressive treatment effectiveness involves systematically measuring drug concentrations in the blood of patients and adjusting dosages accordingly. Additional methods include assessing the function and biopsy of the transplanted organ; however, these methods do not help to predict or detect the risk of early transplant rejection in patients. With advances in biotechnology, the identification of genes and transcripts involved in transplanted organ rejection has begun. The detection of miRNAs or cfDNA levels from dying allograft cells has garnered significant attention [5]. These approaches offer opportunities to personalize the prognosis for CLAD in transplant recipients. However, they still fail to provide definitive guidance on executing effective and functional immunosuppressive therapy for individual patients.

Recent studies have indicated that TTV serves as an effective marker for assessing and monitoring immunocompetence levels, as TTV levels significantly increase in transplant recipients undergoing immunosuppressive treatment [6,7,8,9,10,11,12]. This includes the first prospective cohort study of 143 lung recipients, where over 3000 plasma samples were analyzed to determine TTV DNA presence. A three-year follow-up study revealed a significant association between clinical complications and TTV levels in the blood of the recipients [1]. Research involving liver or kidney transplant recipients revealed an association between lower Alphatorquevirus levels in the blood and organ rejection, suggesting serum TTV levels as biomarkers for the biological effects of immunosuppressive drugs [11,13]. Accordingly, we evaluated TTV load and growth kinetics relative to immunosuppression strength in patients before and after lung transplantation. Our results indicated statistically significant differences in log_10_ TTV according to the time post-transplantation. Before transplantation, the median log_10_ TTV was 3.8 log_10_ copies/mL, with a peak at approximately day 150 at 8.3 log_10_ copies/mL. Comparatively, Jaksch et al. reported a median log_10_ TTV of approximately 4.0 copies/mL in patients before lung transplantation, increasing to a median of 9.6 log_10_ copies/mL by day 143 [1]. Görzer et al. and Gotlieb et al. monitored TTV loads in the first 3 months post-transplantation and reported log_10_ TTV values similar to those at our center. However, differences in assay results may be attributed to the methodology and linearity of the standard curve used in the tests [8]. The immunosuppression scale developed and used in our study provided a means to assess immunosuppression strength, ranging from 2 to 12 units. A positive correlation (ρ = 0.464, *p* < 0.001) between log_10_ TTV and immunosuppression strength was observed, affirming that higher log_10_ TTV values correspond to greater immunosuppression strength. Therefore, the immunosuppression scale is beneficial in elucidating the relationship between treatment, TTV level, and clinical status of the patient.

The authors acknowledge several limitations of this article. This research had a non-prospective character and focused on a retrospective analysis of the study group. Additionally, the small patient sample size and short observation time further limit this study’s scope. The absence of propensity score matching and multifactorial analysis incorporating medical data also restricts the generalizability of the findings. Despite these limitations, our results highlight the potential of TTV as a promising parameter in lung transplant recipients, aiding in the prediction of immunosuppression efficacy and the assessment of organ rejection risk. With this in mind, we decided to publish our study to contribute to the field of transplantology and encourage further research in this area.

## 5. Conclusions

TTV, which was previously unassociated with any disease and characterized by its ubiquitous nature, may serve as a promising marker of immunosuppression levels due to its altered viral load kinetics in solid-organ transplant recipients. Our findings revealed statistically significant differences in log_10_ TTV over time post-transplantation and an increase up to approximately day 150. A positive correlation between log_10_ TTV and immunosuppression strength was established. These findings substantiate the feasibility of using serum TTV levels in lung transplant recipients to help predict the efficacy of ongoing immunosuppressive therapy.

## Data Availability

The data presented in this study is available on request from the corresponding author.
